# Absolute quantification of mammalian microRNAs for therapeutic RNA cleavage and detargeting

**DOI:** 10.1261/rna.080566.125

**Published:** 2025-08

**Authors:** Carolyn Kraus, Jiayi Wang, Haiying Zheng, Jennifer Broderick, Nandagopal Ajaykumar, Mina Zamani, Mengqi Yang, Katharine Cecchini, Shun-qing Liang, Olena Kolumba, Kathryn Chase, Jooyoung Lee, Wen Xue, Erik J. Sontheimer, Ildar Gainetdinov

**Affiliations:** 1 RNA Therapeutics Institute, University of Massachusetts Chan Medical School, Worcester, Massachusetts 01655, USA; 2 Department of Biology, New York University, New York, New York 10003, USA; 3 Division of Gastroenterology, Hepatology, and Nutrition, University of Minnesota Twin Cities, Minneapolis, Minnesota 55455, USA

**Keywords:** absolute abundance, detargeting, microRNA, tissue-specific expression

## Abstract

MicroRNAs (miRNAs) are small regulatory RNAs that destabilize partially complementary transcripts and cleave perfectly paired targets. miRNAs are often expressed in a specific tissue, allowing miRNA-directed cleavage to be used to prevent genome editing or gene replacement therapy in unintended cell types, a strategy called detargeting. miRNA intracellular concentration influences the potency of gene silencing, yet the absolute steady-state levels of just a few miRNAs have been determined in mammalian tissues. Here, we report the absolute abundance of miRNAs in 14 human and mouse cell lines and 17 mouse tissues, including eight brain regions. Our experiments in human cultured cells demonstrate that both miRNA and target levels influence efficacy of cleavage of fully complementary transcripts. We report the miRNA concentration required for productive cleavage of highly expressed transcripts and identify mouse miRNAs that reach this threshold in vivo.

## INTRODUCTION

In plants and animals, microRNAs (miRNAs, ∼22 nt) direct Argonaute proteins to complementary transcripts to regulate host gene expression (Bartel 2018). The ancestral mode of target regulation by Argonautes is cleavage of transcripts that are fully complementary to the small RNA (Hammond et al. 2001; Hutvágner and Zamore 2002; Martinez et al. 2002). For example, plant and cnidarian miRNAs guide Argonautes to cleave nearly perfectly complementary targets (Moran et al. 2017; Meyers and Axtell 2019). In animals, several miRNAs also direct the cleavage of extensively complementary transcripts (Yekta et al. 2004; Hansen et al. 2011; Kleaveland et al. 2018). However, most animal miRNAs bind targets via just 7 nt pairing (5′ seed) and recruit deadenylation complexes to destabilize or repress the translation of target mRNAs (Jonas and Izaurralde 2015; Bartel 2018).

Gene regulation by miRNAs is required for diverse biological processes, including embryogenesis, tissue differentiation, fertility, and immune responses (Wystub et al. 2013; Chen et al. 2014; Parchem et al. 2015; Pua et al. 2016; Bartel 2018; Yuan et al. 2019; Gutiérrez-Pérez et al. 2021). miRNAs are often present in one or just a few tissues (Lagos-Quintana et al. 2002; Landgraf et al. 2007; Liang et al. 2007; Hausser et al. 2009; Díaz-Prado et al. 2012; Ludwig et al. 2016; Smith et al. 2016; de Rie et al. 2017; McCall et al. 2017; Isakova et al. 2020). Such cell-type-specific miRNA expression is used to confine target silencing to a subset of tissues (Brown et al. 2007; Xie et al. 2011; Hirosawa et al. 2019; Hoffmann et al. 2019; Wang et al. 2019; Garcia-Guerra et al. 2025). For example, (1) introducing sites for liver- and muscle-specific miRNAs enables repression of recombinant adeno-associated virus (rAAV) transgenes in these tissues without affecting their expression in the central nervous system (Xie et al. 2011); (2) rAAV transcripts containing miR-142 binding sites are silenced in dendritic cells, which reduces the cytotoxic T-cell response upon rAAV injection (Xiao et al. 2019); and (3) cleavage of the rAAV transcript encoding an anti-CRISPR protein and bearing miR-122 target sites results in Cas9 activation and genome editing restricted to the liver (Lee et al. 2019).

miRNA intracellular concentration dictates silencing efficacy (Mullokandov et al. 2012; Bosson et al. 2014; Denzler et al. 2014; Brancati and Großhans 2018), but the absolute levels are known for just a few miRNAs (Bissels et al. 2009; Xie et al. 2011; Bosson et al. 2014; Denzler et al. 2014). Here, we measured the absolute miRNA abundance in mammalian tissues and cell lines. Our experiments in human cells show that cleavage efficacy of fully complementary targets is determined by both miRNA and target concentrations. We define the miRNA abundance threshold necessary for productive cleavage of highly expressed transgenes. Together, these data provide a quantitative view of miRNA expression across mammalian tissues and will serve as a resource for designing gene therapy and gene-editing strategies with tissue-specific expression.

## RESULTS

### Accurate measurement of absolute miRNA abundance using deep sequencing

Hybridization-, quantitative PCR–, and sequencing-based methods are used to measure miRNA abundance (Lagos-Quintana et al. 2002; Baskerville and Bartel 2005; Ason et al. 2006; Landgraf et al. 2007; Liang et al. 2007; Bissels et al. 2009; Díaz-Prado et al. 2012; Mestdagh et al. 2014; Ludwig et al. 2016; Smith et al. 2016; de Rie et al. 2017; McCall et al. 2017; Godoy et al. 2019; Isakova et al. 2020). A caveat of small RNA high-throughput sequencing is that adapter ligation efficiency varies for different small RNAs, and such variation results in up to 1000-fold distortion of the relative abundance of miRNAs in the sequencing data (Hafner et al. 2011; Fuchs et al. 2015; Giraldez et al. 2018; Kim et al. 2019). The ligation bias can be minimized by driving reactions to completion using (1) extended incubation time, (2) randomized terminal sequences in adapter oligonucleotides, and (3) PEG-8000 to increase the effective reactant concentration (Giraldez et al. 2018; Godoy et al. 2019; Kim et al. 2019).

We incorporated these modifications when sequencing an equimolar mix of 36 synthetic RNAs (20–30 nt in length) and recovered all sequences with ≤5-fold distortion of their relative abundance: ∼0.2–2.3 times of the expected read counts (median: 0.96; interquartile range [IQR]: 0.55–1.42; relative standard deviation: <10% for three independent trials; Supplemental Table S1; Supplemental Fig. S1A). Our results were consistent with the prior benchmarking of small RNA sequencing methods that used a pool of 962 synthetic small RNAs (Supplemental Table S2; Supplemental Fig. S1B; Giraldez et al. 2018). Compared to commercially available kits, our in-house method that uses both randomized adaptors and PEG-8000 was least biased: for most synthetic RNAs, the observed-to-expected ratio of sequencing reads was close to 1 (median: 0.81; IQR: 0.4–1.4) for the improved protocol, compared to 0.09 (IQR: 0.01–0.50) for TruSeq and 0.03 (IQR: 0.00–0.34) for NEBNext (Supplemental Table S2; Supplemental Fig. S1B).

**FIGURE 1.**
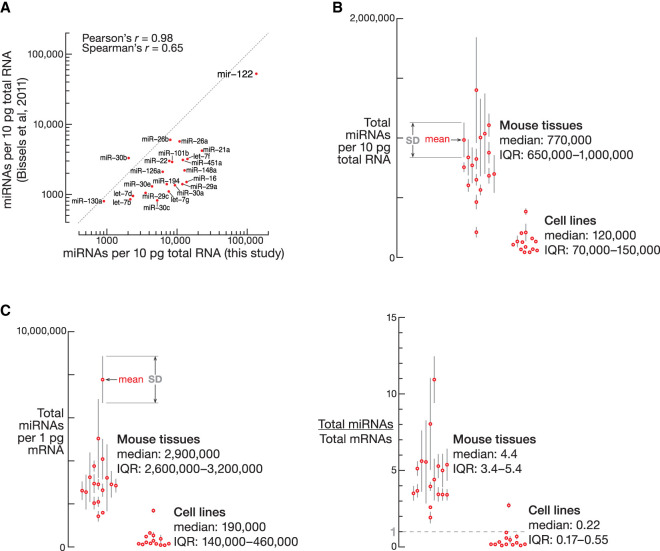
Measurement of absolute abundance of miRNAs. (*A*) miRNA abundance in the mouse liver measured with a hybridization-based method (Bissels et al. 2009) and deep sequencing (this study). (*B*) Absolute abundance of all miRNAs per total RNAs in mouse tissues and mouse and human cell lines (numerical data are in Supplemental Table S3). (*C*) Absolute abundance of all miRNAs per total mRNAs in mouse tissues and mouse and human cell lines (numerical data are in Supplemental Table S4).

To measure the absolute abundance of miRNAs in animal tissues, we selected nine synthetic small RNAs that did not match the human, mouse, fly, or worm genomes (Supplemental Table S1; mean observed-to-expected ratio: 0.75). We added the nine-oligonucleotide pool to total RNA before preparing sequencing libraries from immortalized cell lines and mouse tissues (Supplemental Table S3; Supplemental Fig. S2). Our data from mouse liver were comparable to prior quantification with a microarray-based approach (Fig. 1A; Pearson's *r* = 0.98, *P* = 1 × 10^−16^; Spearman's *r* = 0.65, *P* = 0.001; Bissels et al. 2009). For example, we estimated miR-122 abundance at 140 ± 20 × 10^3^ molecules/10 pg total RNA (*n* = 3), and the microarray method reported 50 ± 10 × 10^3^ miR-122 molecules/10 pg total RNA (*n* = 4; Bissels et al. 2009). (A mammalian cell contains ∼10–20 pg total RNA [Han and Lillard 2000; Tang et al. 2011].) Estimates of miRNA abundance obtained with our deep sequencing approach were on average ∼3.4-fold higher than those from microarrays (Fig. 1A).

### Absolute abundance of miRNAs in 14 cell lines and 17 mouse tissues

To compare absolute miRNA levels among cell types, we measured their abundance in 14 cell lines (fully confluent cultures; Hwang et al. 2009) and 17 mouse tissues, including eight regions of the brain and three regions of intestines (*n* = 2–6; Supplemental Table S3; Supplemental Fig. S2). The total miRNA abundance ranged 33-fold, from 43 ± 8 × 10^3^ miRNAs per 10 pg total RNA in K562 (*n* = 2) and HepG2 (*n* = 2) cells to 1100 ± 100 × 10^3^ (*n* = 4) in the heart and 1400 ± 400 × 10^3^ (*n* = 3) in the skeletal muscle (Fig. 1B; Supplemental Table S3). As observed previously (Lu et al. 2005; Gaur et al. 2007), tissues contained more miRNAs compared to cultured cells. The median total miRNA abundance was ∼120,000 (IQR: 70,000–150,000) per 10 pg total RNA in cell lines versus ∼770,000 (IQR: 650,000–1,000,000) in mouse tissues (Mann–Whitney test, *P* = 7 × 10^−9^; Fig. 1B; Supplemental Table S3).

We determined the fraction of mRNAs in each sample by sequencing total RNA without depleting rRNAs (Supplemental Table S4; Supplemental Fig. S1C). Our analyses showed that miRNA-to-mRNA ratio was also higher in the animal tissues: median miRNA-to-mRNA molar ratio was 0.22 (IQR: 0.17–0.55) in cultured cells versus 4.4 (IQR: 3.4–5.4) in mouse tissues (Fig. 1C; Supplemental Table S4).

Consistent with the higher total miRNA levels in animal organs, we find that the steady-state abundance of known tissue-specific miRNAs is greater in animal samples than in the corresponding cell lines. For example, miR-122 (5′-UGGAGUGUGACAAUGGUGUUU) is 14-fold more abundant in liver (∼140,000/10 pg total RNA) than in Huh-7.5 cells (∼10,000/10 pg total RNA; Supplemental Table S5). The level of miR-1a (5′-UGGAAUGUAAAGAAGUAUGUA) increased from 65 ± 5 to 9000 ± 500 copies per 10 pg total RNA in C2C12 cells upon their differentiation to myotubes (Supplemental Table S5), yet miR-1a abundance was ∼31-fold higher in the heart (280,000 ± 20,000/10 pg total RNA) and ∼78-fold higher in skeletal muscle (700,000 ± 200,000/10 pg total RNA; Supplemental Table S5).

### Target and miRNA steady-state levels determine the efficacy of transcript cleavage

**FIGURE 2.**
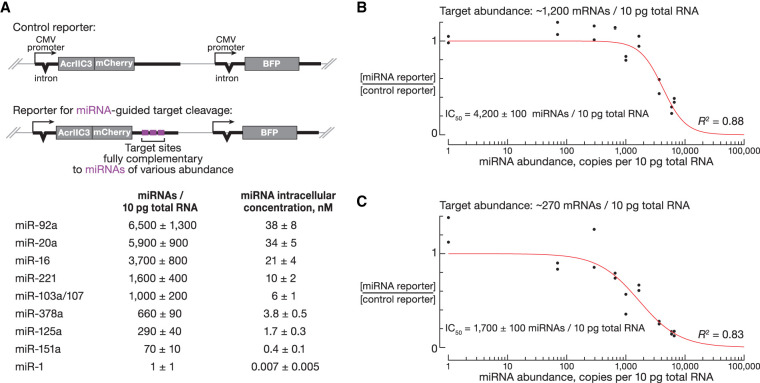
miRNA absolute abundance predicts silencing efficacy of fully complementary targets. (*A*) Reporters with fully complementary target sites to miRNAs of different abundance. Blue fluorescent protein (BFP) is used to measure the fraction of transfected HEK293T cells. (*B*) Relative and absolute abundance of reporter mRNAs with perfectly complementary target sites to miRNAs of different intracellular concentration (*n* = 2); 100 ng of plasmid DNA was used for each transfection of 150,000 HEK293T cells (numerical data are in Supplemental Table S6). (*C*) Relative and absolute abundance of reporter mRNAs with perfectly complementary target sites to miRNAs of different intracellular concentration (*n* = 2); 10 ng of plasmid DNA was used for each transfection of 150,000 HEK293T cells (numerical data are in Supplemental Table S6).

Intracellular miRNA concentration influences the extent of target silencing via miRNA seed pairing (Mullokandov et al. 2012; Denzler et al. 2014; Brancati and Großhans 2018). To identify endogenous miRNAs suitable for therapeutic RNA cleavage, we sought to determine the absolute miRNA abundance required for productive target cleavage. HEK293T cells were transfected with plasmids encoding reporter mRNAs with 3′ UTRs containing target sites fully complementary to miRNAs of various abundance, ranging from 6600 ± 1300 miRNAs (miR-92a) to 1 ± 1 miRNAs per 10 pg total RNA (miR-1; Fig. 2A). The control reporter lacked perfect pairing to any HEK293T miRNA. Each reporter plasmid also expressed blue fluorescent protein from a separate promoter allowing for measurement of the fraction of transfected cells with flow cytometry (Fig. 2A). Following transfections, total RNA was extracted, and poly(A)+ transcripts were sequenced. A pool of 92 synthetic mRNAs (ERCC RNA Spike-In Mix, see Materials and Methods) was added before poly(A)+ selection and preparing sequencing libraries to enable absolute quantification of reporter and endogenous mRNA abundance in transfected cells.

Reporters with target sites for abundant miRNAs were repressed to a greater degree, compared to reporters with complementarity to less abundant miRNAs (Fig. 2B; Supplemental Table S6). The relationship between miRNA abundance and reporter repression efficiency followed the inhibitor dose–response curve with the half maximal inhibitory concentration (IC_50_) of 4200 ± 100 miRNAs per 10 pg total RNA (*R*^2^ = 0.88; Fig. 2B). In transfections with no detectable repression, the median steady-state level of reporter mRNA was 1200 copies per 10 pg total RNA (IQR: 1000–1700; *n* = 10; Supplemental Table S6), comparable to that of the endogenous *GAPDH* (median: 990; IQR: 940–1050; *n* = 36) and *EEF1A1* mRNAs (median: 1500; IQR: 1400–1700; *n* = 36; Supplemental Table S7).

To determine the miRNA concentration sufficient to silence mRNAs of lower abundance, we repeated transfection experiments with smaller amounts of reporter plasmids. When HEK293T cells were transfected with less plasmid DNA (see Materials and Methods), the median reporter mRNA abundance was 270 copies per 10 pg total RNA (IQR: 230–570; *n* = 10; Supplemental Table S6), similar to that of *PABPC1* (median: 240; IQR: 230–260) and *ATF4* mRNAs (median: 320; IQR: 310–360; Supplemental Table S7). Compared to the IC_50_ of 4200 ± 100 miRNAs per 10 pg total RNA for the reporter at 1200 copies per 10 pg total RNA (Fig. 2B), IC_50_ was 1700 ± 100 miRNAs for the 270-copy reporter mRNA (Fig. 2C; Supplemental Table S6).

Our results thus establish the miRNA concentration threshold for productive silencing of highly expressed transgenes. These data also demonstrate that both miRNA and target steady-state levels predict the efficacy of gene repression via cleavage of fully complementary sites (Mukherji et al. 2011; Mullokandov et al. 2012; Brancati and Großhans 2018).

### miRNAs for tissue-specific cleavage of highly expressed transcripts

Many miRNAs are expressed only in a subset of cell types (Landgraf et al. 2007; Zhao et al. 2007; Liu and Olson 2010; Dai et al. 2016; Ludwig et al. 2016; Smith et al. 2016; de Rie et al. 2017; McCall et al. 2017; Bushel et al. 2018; Isakova et al. 2020). To identify tissue-specific miRNAs with absolute abundance sufficient to productively cleave highly expressed transgenes, we used the concentration cutoff established in our HEK293T transfection experiments (1700 miRNAs/10 pg total RNA; Fig. 2B; Supplemental Table S6). In our analyses, we surveyed all miRNA isoforms including those with distinct 5′ ends (5′-iso-miRs) or produced from the nondominant arm of the pre-miRNA (miRNAs*, also known as passenger strands).

Our search criteria (>1700 copies per 10 pg total RNA in one tissue and <340 copies in all other tissues) were satisfied for many known tissue-specific miRNAs and some of their iso-miRs (miR-1a, miR-122, miR-133a, and miR-215; Supplemental Tables S5, S8; Landgraf et al. 2007; Zhao et al. 2007; Liu and Olson 2010; Dai et al. 2016). The brain expresses the largest number of tissue-specific miRNAs (Landgraf et al. 2007; Ludwig et al. 2016; Smith et al. 2016; de Rie et al. 2017; McCall et al. 2017; Bushel et al. 2018; Isakova et al. 2020). Yet the levels of only four miRNAs met our abundance requirements (miR-9/124/127/138 and their iso-miRs; Supplemental Table S8). In fact, several miRNAs previously reported as brain-specific were present at <200 copies per 10 pg total RNA in all brain tissues (e.g., miR-878 and miR-666; Supplemental Table S5) and are thus unlikely to elicit efficient silencing. Strikingly, the eight regions of the brain were nearly identical in their miRNA expression profiles (Supplemental Table S5; Supplemental Fig. S3). Only miR-193b and miR-206 were expressed in just one or two parts of the brain. miR-193b (5′-AACUGGCCCACAAAGUCCCGC) was present at ∼500–600 copies/10 pg total RNA in the caudate putamen and nucleus acumbens, <200 copies in the thalamus and skeletal muscle, and <100 copies in all other tissues (Supplemental Table S5). The abundance of miR-206 (5′-UGGAAUGUAAGGAAGUGUGUG) was ∼600 copies/10 pg total RNA in the cerebellum but also ∼2300 in the skeletal muscle (Supplemental Table S5; Panwalkar et al. 2015).

We also identified miRNAs with absolute levels at >1700 copies per 10 pg total RNA in ≤3 (Supplemental Table S9A) or in ≤5 tissues (Supplemental Table S9B). For the applications that require a combination of two miRNAs to be tissue-specific (e.g., Wang et al. 2019), we compiled a list of four such miRNA pairs coexpressed exclusively in the cerebellum, kidney, or skeletal muscle (Supplemental Table S10). Finally, our data allowed finding miRNAs suitable for transgene detargeting. For example, miR-181a (5′-AACAUUCAACGCUGUCGGUGA) and miR-23b (5′-AUCACAUUGCCAGGGAUUACC) should be usable for efficient detargeting of transgenes in all tissues except liver and spleen, respectively: miR-181a and miR-23b are expressed at >1000 copies/10 pg total RNA in all organs, but at <300 copies in liver and spleen, respectively (Supplemental Table S5).

## DISCUSSION

Tissue-specific expression of miRNAs permits their use for the control of transgene expression (Brown et al. 2007; Wang et al. 2019). Here, we used a minimally biased sequencing approach combined with external spike-in standards to enable accurate measurement of absolute miRNA abundance in mammalian cells and tissues (Supplemental Table S5).

Normalized to mRNAs or total RNAs, animal tissues were found to contain several-fold higher levels of miRNAs than cell lines (Fig. 1B,C). Perhaps, the extraordinary stability of miRNAs (Kingston and Bartel 2019; Reichholf et al. 2019) contributes to such difference in their abundance: miRNAs may accumulate to greater levels in the nondividing cells in postnatal organs compared to the proliferating immortalized cell lines (Kingston and Bartel 2019). Because miRNA abundance dictates the degree of target repression, genetic ablation of miRNAs is expected to produce overlapping but distinct transcriptomic changes in immortalized cells and in vivo.

Consistent with previous reports (Mukherji et al. 2011; Mullokandov et al. 2012; Brancati and Großhans 2018), we find that both miRNA and mRNA levels determine the extent of target repression. We determined the minimal miRNA concentration required for efficient cleavage of abundant mRNAs. This threshold enabled identification of endogenous miRNAs predicted to productively cleave highly expressed transcripts in mouse tissues. Our data will facilitate the design of gene replacement therapies and genome editing with targeted expression.

## MATERIALS AND METHODS

### Mice

Mice (C57BL/6J, IMSR # JAX:000664) were housed in an Association for Assessment and Accreditation of Laboratory Animal Care International–accredited barrier facility at controlled temperature (22°C ± 2 °C), relative humidity (40% ± 15%) and a 12 h day–light cycle. All experimental animals were 4–6 months old. All procedures were reviewed and performed in compliance with the guidelines of the Institutional Animal Care and Use Committee (IACUC) of New York University (protocol number 2024-1207) and the University of Massachusetts Chan Medical School (protocol number PROTO202000051).

### Cell lines

Immortalized cultured cells were maintained as described in Supplemental Table S11.

### Reporter experiments in HEK293T cells

HEK293T cells were grown in DMEM (Fisher, 11965092) supplemented with 10% FBS (Fisher, 16000044) at 37°C in 5%CO_2_. HEK293T cell volume (∼1150 µm^3^) was calculated based on their median diameter (∼13 µm) measured with phase contrast microscopy (Leica DMi8). For each transfection, a “filler” plasmid without the reporter (p2-M427 + pIRESpuro; a gift from the Wolfe lab, UMass Chan Medical School) was added to 10 or 100 ng of the reporter to keep the total amount of transfected DNA at 200 ng. HEK293T cells at ∼70% confluency were trypsinized, diluted to 3 × 10^5^ cells/mL in full media, and 500 µL of cell suspension (1.5 × 10^5^ cells) was added to 4 µL water containing plasmids. Next, 2 µL PolyFect (QIAGEN, #301105) mixed with 18 µL serum-free media was added to the cells and plasmids from the previous step. Cells were incubated at room temperature for 10 min, and then each transfection was plated into a single well of a 24-well plate; 72 h post-transfection, cells were trypsinized, diluted in 1000 µL PBS, centrifuged at 500*g*, and pellets were washed in 1000 µL PBS. Next, a 500 µL aliquot was centrifuged at 500*g*, and pellets snap-frozen in liquid nitrogen for RNA extraction and RNA sequencing. The other 500 µL of the cell suspension was analyzed on a Miltenyi MACSQuant VYB Benchtop Analyzer to measure the total number of cells and the fraction of transfected cells. Briefly, the 488 nm laser was used to record forward and side scatter to select single cells. The 405 nm laser was used to excite BFP. BFP emission was detected using a 452/45 nm bandpass filter. Fraction of transfected cells in Supplemental Table S6 was measured using BFP fluorescence against the nontransfected control.

### Small RNA-seq library preparation

Total RNA was extracted using the mirVana miRNA Isolation Kit (Fisher, AM1560). Small RNA libraries were constructed as described (Gainetdinov et al. 2021). For the 36-oligonucleotide pool, 100 fmol (10 µL of 10 nM pool) was used for each replicate (*n* = 3). For library preparation using RNA from cell lines and animal tissues, an equimolar mix of nine synthetic spike-in RNA oligonucleotides was added to each RNA sample to enable absolute quantification of small RNAs (Supplemental Table S3). To reduce ligation bias and eliminate PCR duplicates, the 3′ and 5′ adaptors both contained nine random nucleotides at their 5′ and 3′ ends, respectively (Fu et al. 2018), and ligation reactions contained 25% (w/v) PEG-8000 (f.c.). Briefly, total RNA was first ligated to 25 pmol of 3′ DNA adapter with adenylated 5′ and dideoxycytosine-blocked 3′ ends (5′-/rApp/NNNGTCNNNTAGNNNTGGAATTCTCGGGTGCCAAGG/ddC/) in 30 µL of 50 mM Tris-HCl (pH 7.5), 10 mM MgCl_2_, 10 mM DTT, and 25% (w/v) PEG-8000 (NEB) with 600U of T4 Rnl2tr K227Q (homemade) at 16°C overnight. After ethanol precipitation, the 50–90 nt (14–54 nt small RNA + 36 nt 3′ UMI adapter) 3′ ligated product was purified from a 15% denaturing urea-polyacrylamide gel (National Diagnostics). After overnight elution in 0.4 m NaCl followed by ethanol precipitation, the 3′ ligated product was denatured in 14 µL water at 90°C for 60 sec, and 1 µL of 50 µM RT DNA primer (5′-CCTTGGCACCCGAGAATTCCA) was added and annealed at 65°C for 5 min to suppress the formation of 5′-adapter:3′-adapter dimers during the next step. The resulting mix was then ligated to a mixed pool of equimolar amount of two 5′ RNA adapters (to increase nucleotide diversity at the 5′ end of the sequencing read: 5′-GUUCAGAGUUCUACAGUCCGACGAUCNNNCGANNNUACNNN and 5′-GUUCAGAGUUCUACAGUCCGACGAUCNNNAUCNNNAGUNNN) in 20 µL of 50 mM Tris-HCl (pH 7.8), 10 mM MgCl_2_, 10 mM DTT, 1 mM ATP, and 25% (w/v) PEG-8000 (NEB) with 20U of T4 RNA ligase (Fisher, AM2141) at 25°C for 2 h. The ligated product was precipitated with ethanol, cDNA synthesis was performed in 20 µL at 42°C for 1 h using AMV reverse transcriptase (NEB, M0277), and 5 µL of the RT reaction was amplified in 25 µL using AccuPrime *Pfx* DNA polymerase (Fisher, 12344024; 95°C for 2 min, 15 cycles of: 95°C for 15 sec, 65°C for 30 sec, and 68°C for 15 sec; forward primer sequence: 5′-A ATGATACGGCGACCACCGAGATCTACACGTTCAGAGTTCTACAGTCCGA; reverse primer sequence: 5′-CAAGCAGAAGACGGCATACGAGATXXXXXXGTGACTGGAGTTCCTTGGCACCCGAGAATTCCA, where XXXXXX represent 6 nt sequencing barcode). The PCR product was purified in a 2% agarose gel. Small RNA-seq libraries samples were sequenced using a NextSeq 500 (Illumina) to obtain 79 nt, single-end reads.

### Analysis of small RNA data sets

The 3′ adapter (5′-TGGAATTCTCGGGTGCCAAGG-3′) was removed with cutadapt (v4.1), PCR duplicates were eliminated as described (Fu et al. 2018), and rRNA matching reads were removed with bowtie (parameter -v 1; v1.0.0; Langmead et al. 2009) against *Mus musculus* or *Homo sapiens* set in SILVA rRNA database (Glöckner et al. 2017). For further analyses, we considered only reads that mapped in the sense orientation to miRNA hairpin loci without mismatches or with a single-nucleotide mismatch at the miRNA 3′ end to account for nontemplated addition of 3′ terminal nucleotides (miRbase was downloaded in December 2023; Kozomara et al. 2019). miRNA reads with the same 5′ end were grouped to represent a single 5′ isomiR, and each 5′ isomiR was analyzed separately. Sequences of synthetic spike-in oligonucleotides (Supplemental Table S5) were identified allowing no mismatches (bowtie parameter -v 0; v1.0.0; Langmead et al. 2009), and the absolute abundance of miRNAs was calculated as follows (see “spikein_added” and “total_RNA_added” values in Supplemental Table S3):

$$\begin{array}{rl} & \mathrm{Molecules}\;\mathrm{per}\;10\;\mathrm{pg}\;\mathrm{total}\;\mathrm{RNA}=\\ & \frac{\mathrm{miRNA}\_\mathrm{reads}\times \mathrm{spikein}\_\mathrm{added}\;(\mathrm{mol})\times 6.022\times 10^{23}\times 10}{\mathrm{spikein}\_\mathrm{reads}\times \mathrm{total}\_\mathrm{RNA}\_\mathrm{added}\;(\mathrm{pg})} \end{array}$$

### RNA-seq library preparation

Total RNA was extracted using mirVana miRNA Isolation Kit (Thermo Fisher, AM1560). RNA-seq of total RNAs without rRNA depletion (Supplemental Table S3) was performed with NEBNext UltraExpress RNA Library Prep Kit (NEB, #E3330S), except that UMI-containing adaptors were used (Fu et al. 2018). For sequencing of polyadenylated RNAs (Supplemental Table S6), NEBNext Poly(A) mRNA Magnetic Isolation Module (NEB, #E7490S) was used; to enable absolute quantification of RNAs, before library preparation, 1 µL of 1:100 diluted ERCC spike-in mix 1 (Thermo Fisher, 4456740) was added to total RNA (Supplemental Table S6). RNA-seq libraries were sequenced using an AVITI benchtop sequencer (Element Biosciences) to obtain 150 + 150 nt, paired-end reads.

### Analysis of RNA-seq data

RNA-seq analysis was performed as described (Gainetdinov et al. 2021). Briefly, sequences were reformatted to extract unique molecular identifiers (Fu et al. 2018); the reformatted reads were then aligned to rRNA using bowtie2 (v2.2.0; Langmead and Salzberg 2012). Unaligned reads were mapped to mouse (mm10) or human (hg38) genome using STAR (v2.3.1; Dobin et al. 2013), and PCR duplicates were removed (Fu et al. 2018). To account for the bias against rRNA sequencing reads near modified nucleotides, the rRNA adjustment coefficient was calculated for each sample (i.e., the mean coverage divided by the maximum coverage detected across all rRNA genes). Total number of mRNA mapping reads was calculated using BEDTools (v2.29.2; Quinlan and Hall 2010) by intersecting alignments with protein-coding genes on the same strand. Transcript abundance was calculated using StringTie (v1.3.4; Pertea et al. 2016). Mean mRNA length in Supplemental Table S3 was calculated as:

$$\mathrm{Mean}\;\mathrm{mRNA}\;\mathrm{length}=\frac{\sum_{i=1}^{n}{\mathrm{mRNA\_length}}_{i}\times {\mathrm{mRNA\_fpkm}}_{i}}{\sum_{i=1}^{n}{\mathrm{mRNA\_fpkm}}_{i}}$$

IC_50_ was determined by fitting the following equation to data from Supplemental Table S6:

$$\mathrm{Repression}=\;\frac{1}{1+e^{\mathrm{slope}\times ({\mathrm{IC}}_{50}-\mathrm{miRNA\_concentration})}}$$

The fit was performed using the Trust Region Reflective algorithm implemented in the optimize.curve_fit function from Python module scipy (v.1.8.1) for the maximum number of 10,000 function evaluations before the termination. The following physically meaningful constraints on the parameters were used: −100 ≤ slope ≤ 0; 1 ≤ IC_50_ ≤ 10,000 miRNAs per 10 pg total RNA.

## DATA DEPOSITION

Sequencing data are available from the National Center for Biotechnology Information Small Read Archive using accession number PRJNA1140118.

## SUPPLEMENTAL MATERIAL

Supplemental material is available for this article.

## COMPETING INTEREST STATEMENT

E.J.S. is a cofounder and Scientific Advisory Board member of Intellia Therapeutics and a Scientific Advisory Board member at Tessera Therapeutics. The remaining authors declare no competing interests.

## Figures and Tables

**Carolyn Kraus:**
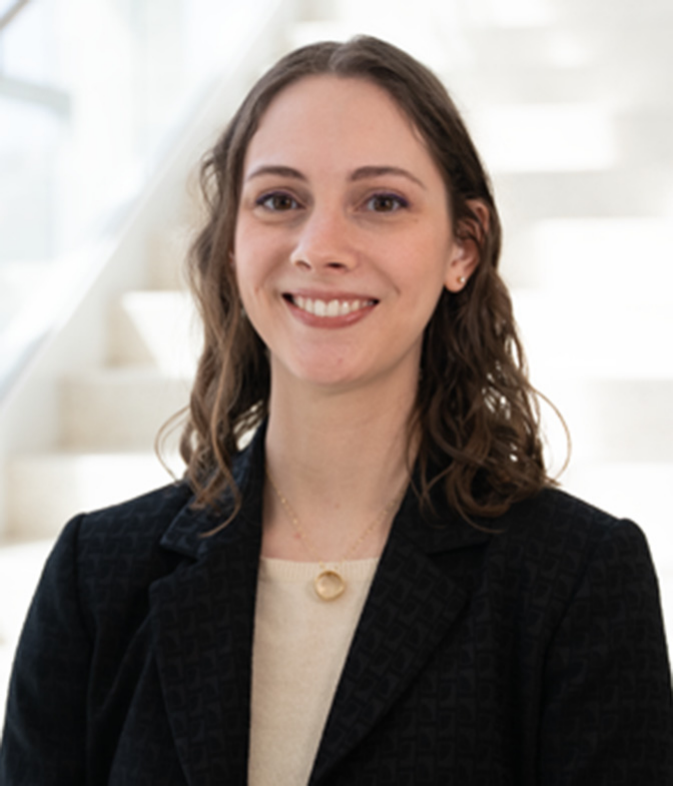


**Jiayi Wang:**
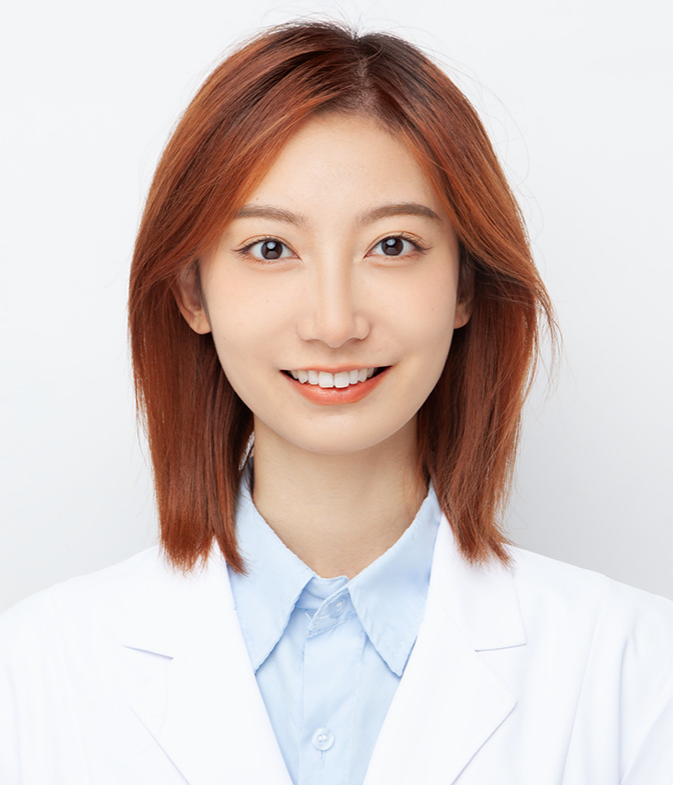


## MEET THE FIRST AUTHORS

***Meet the First Author(s)* is an editorial feature within *RNA*, in which the first author(s) of research-based papers in each issue have the opportunity to introduce themselves and their work to readers of *RNA* and the RNA research community. Carolyn Kraus and Jiayi Wang are co-first authors of this paper, “Absolute quantification of mammalian microRNAs for therapeutic RNA cleavage and detargeting.” Carolyn is a graduate student in the laboratory of Erik Sontheimer at the RNA Therapeutics Institute of the University of Massachusetts Chan Medical School in Worcester. Her main research focus is applications of CRISPR/Cas9 gene editing and anti-CRISPRs. Jiayi did this work in the Sontheimer lab, and is now a postdoc at the Center of Infectious Diseases, Division of Infectious Diseases in State Key Laboratory of Biotherapy, West China Hospital, Sichuan University, Chengdu, Sichuan, China. Jiayi's research focuses on the biological processes of viral transcription and replication, and applications of CRISPR.**

**What are the major results described in your paper and how do they impact this branch of the field?**

**CK:** In this paper, we publish an impressive collection of microRNA sequencing data sets from many mouse tissues and human or mouse cell lines. With these data sets, we were able to experimentally show that microRNA abundance (as calculated from these data sets) correlates with the ability to repress a transgene. These sequencing data sets highlight differences between mouse tissues, and they also highlight differences in microRNA expression between cell lines and tissues to provide valuable insight into how researchers may best design translational and basic microRNA studies in the future.

**JW:** The major results of our paper demonstrate that the intracellular concentration of miRNAs directly predicts the efficacy of silencing fully complementary target transcripts. The finding advances our understanding of miRNA biology by providing quantitative insights into miRNA abundance and stability, thereby informing the design of miRNA-based therapies and enriching this branch of RNA regulation research.

**What led you to study RNA or this aspect of RNA science?**

**CK:** I was naturally drawn to work with RNA-based systems like microRNA and CRISPR/Cas9 because I enjoy tinkering and creating new technologies from existing systems found in nature. Learning that endogenous microRNAs can be used to control a transgene was fascinating to me! And testing how well different microRNAs can repress a transgene was a natural next step in our research.

**JW:** The precise quantification of miRNAs in different tissues represented an important gap in the field, and I wanted to contribute to filling that knowledge gap to improve RNA-based therapeutic strategies. Additionally, the discovery that miRNA profiles vary across different organs/tissues suggests their potential as natural tissue-specific tools, which could be harnessed for future clinical applications.

**What are some of the landmark moments that provoked your interest in science or your development as a scientist?**

**CK:** I've always had a passion for biology, but my interest in research began in high school during a field trip where we heard someone speak about how the small molecule kinase inhibitor Gleevec was developed, and how it changed the face of cancer treatment. During college, when I first began working at the bench, I remember the discovery of CRISPR/Cas9 as an RNA-guided nuclease and how exciting it was! Now I'm lucky enough to work with CRISPR/Cas9 full time as a graduate student and it feels like a very satisfying full-circle progression.

**If you were able to give one piece of advice to your younger self, what would that be?**

**JW:** Engage more proactively in scientific communication and seek professional guidance without hesitation. Timely and open exchange—both within the research group and with more experienced colleagues in the broader scientific community—can significantly enhance problem-solving, refine research directions, and accelerate personal and professional growth.

**Are there specific individuals or groups who have influenced your philosophy or approach to science?**

**CK:** All of my past and present research mentors have instilled different lessons about how to approach science. My undergraduate research mentor taught me about looking for different solutions to a research question, my experience working in a laboratory to develop antibody reagents for primate research taught me how to make decisions based on data and move projects forward effectively, and my graduate school mentors have taught me to be confident learning new techniques and trying new things to get to the bottom of my research question. I am especially grateful for my graduate PI, Dr. Sontheimer, for his stalwart and unwavering support and for other exceptional faculty on campus that I turn to for professional and personal guidance, including Drs. Kathleen Engelmann, Athma Pai, Mary Pickering, and many, many more.

**JW:** I received my initial scientific training in the laboratory of Dr. Erik J. Sontheimer, where I was first introduced to the principles of rigorous research. The team's strong culture of collaboration and open resource sharing helped eliminate information gaps and facilitated effective communication, which was essential for advancing our projects.

**What are your subsequent near- or long-term career plans?**

**CK:** As I approach the completion of my PhD, I find myself interested in exploring some new and interesting applications of my scientific training, including medical or scientific writing. Working at the bench will always be my passion, but I am excited to explore something new.

**What were the strongest aspects of your collaboration as co-first authors?**

**CK:** The trust and mutual respect between us was clear when I first started working with Jiayi—I was a brand new graduate student in the laboratory, and she had been working with this project for almost a year before I met her. She was open and helpful from the start and designed great experiments that I was able to build on after she moved on from the laboratory that culminated in this manuscript.

**JW:** The strongest aspects of our collaboration as co-first authors were open communication, complementary skill sets, and mutual respect. Our individual strengths in experimental design, data analysis, and manuscript preparation complemented each other, enhancing the overall quality and pace of the work. Most importantly, a shared commitment to scientific rigor and a collaborative mindset created a productive and supportive working environment.

**How did you decide to work together as co-first authors?**

**JW:** We decided to work together as co-first authors based on our substantial and complementary contributions throughout the course of the project. From the early stages of experimental design to data collection, analysis, and manuscript preparation, we collaborated closely and shared responsibility for all key aspects of the study.
